# The Impact of Depression and Antidepressant Treatment on Patient-Reported Outcomes Following Thumb Carpometacarpal Arthroplasty

**DOI:** 10.1016/j.jhsg.2026.100989

**Published:** 2026-03-24

**Authors:** Omar M. Mohamed, Jessica L. Duggan, Kristen E. Hines, Carl M. Harper, Tamara D. Rozental, Monica M. Shoji

**Affiliations:** ∗ Beth Israel Deaconess Medical Center, Boston, MA; † Harvard Combined Orthopaedic Residency Program, Boston, MA; ‡ Harvard Medical School, Boston, MA

**Keywords:** Arthritis, Depression, Patient-reported outcomes, QuickDASH, Thumb carpometacarpal arthroplasty

## Abstract

**Purpose:**

Depression, a leading cause of disability, is a known predictor of poorer outcomes in upper-extremity procedures. Associated mood and somatic symptoms may interfere with the assessment of postoperative recovery, making it difficult to distinguish between postoperative-related symptoms and those linked to depression. This study examines the impact of depression on patient-reported outcomes following carpometacarpal (CMC) arthroplasty (trapeziectomy with ligament reconstruction and tendon interposition) for basilar thumb arthritis. We hypothesized that patients with a diagnosis of major depressive disorder or active antidepressant treatment before surgery will have poorer upper-extremity outcomes at 1 year compared with patients without depression.

**Methods:**

Patients who underwent isolated thumb CMC arthroplasty at a single academic center were screened for inclusion. Baseline demographic data, psychiatric history, and antidepressant use at the time of surgery were collected retrospectively. Patients with concomitant psychiatric diagnoses other than depression, those undergoing multiple procedures at the time of thumb CMC arthroplasty, or individuals with additional chronic upper-extremity pathology were excluded. The Quick Disability of the Arm, Shoulder, and Hand (*Quick*DASH) questionnaire was administered before surgery to establish baseline scores and at 3, 6, and 12 months after surgery. Quick Disability of the Arm, Shoulder, and Hand scores were analyzed using an independent samples *t* test and a linear mixed effects model to adjust for the time from surgery, sex, race, and body mass index at the time of surgery.

**Results:**

Seventy-three (22.3%) of 328 patients had a diagnosis of depression or were receiving antidepressant treatment at the time of surgery. Before surgery, the average *Quick*DASH score was 41.4 for the patients with depression and 40.3 for patients without. At 1 year after surgery, these averages were 28.8 and 24.0, respectively. No significant difference in *Quick*DASH scores was observed between patients with depression or receiving antidepressant treatment and those without at any time point.

**Conclusions:**

This study demonstrates no significant difference in postoperative *Quick*DASH scores between patients with a diagnosis of depression or antidepressant treatment and those without following thumb CMC arthroplasty. These findings suggest that a history of depression may not adversely affect patient-reported outcomes after thumb CMC arthroplasty.

**Level of evidence:**

Prognostic IV.

Major depressive disorder (MDD) is a common and serious mental health condition that affects an estimated 21 million adults in the United States every year.^1^ Its prevalence continues to rise, with the number of US adults with MDD having increased from 15.5 to 17.5 million (a 12.9% increase) between 2010 and 2018.^2^ Major depressive disorder impact carries consequences that extend well beyond mood symptoms, decreased function, and loss of independence. It has been associated with greater health care costs and lower productivity. The 2017 Global Burden of Disease study found that MDD accounted for the heaviest burden of all mental and behavioral health disorders in adults, representing an estimated 2.7 million disability-adjusted life-years in 2016.^2^^,^^3^

A diagnosis of MDD requires the persistence of at least 5 characteristic symptoms for a minimum of 2 weeks, with at least 1 being depressed mood or loss of interest, resulting in decreased function. These symptoms and functional loss must also not be explained by any other medical or behavioral condition.^4^ The diagnosis is made clinically through a comprehensive clinical and mental status examination, because no objective biomarker exists. Diagnosis of MDD can often be complicated by the overlap of depressive symptoms with chronic illness, pain, and age-related functional decline, which can artificially elevate depression screening scores.^5^ This makes accurate assessment especially challenging in patients with musculoskeletal disorders, where both physical and psychological symptoms often coexist.

In hand surgery, MDD has emerged as an underemphasized risk factor for poorer outcomes. Specifically in hand surgery populations, previous studies have consistently exhibited the deleterious effects that depression has on postoperative pain and patient-reported outcomes.^6^, ^7^, ^8^, ^9^, ^10^, ^11^ For instance, Vranceanu et al^7^ examined 120 patients undergoing minor hand surgeries (such as carpal tunnel release or trigger finger release) and found that preoperative depression was the only independent predictor of both pain intensity and disability after surgery. Additionally, a prevalence study using Patient-Reported Outcomes Measurement Information System (PROMIS) depression measures has shown that patients with thumb carpometacarpal (CMC) arthritis report higher rates of depression, with approximately 1 in 10 screening positive for depressive symptoms.^12^ Several additional studies have correlated the Disabilities of the Arm, Shoulder, and Hand and Quick Disability of the Arm, Shoulder, and Hand (*Quick*DASH) patient-reported outcome surveys with several depression screening scores.^13^, ^14^, ^15^, ^16^ These findings suggest that MDD not only impacts the emotional and mental well-being of patients but also imparts considerable effects on physical recovery following upper-extremity orthopedic procedures.

Thumb CMC arthritis is 1 of the most common sites of osteoarthritis in the upper extremity, resulting in pain, weakness, and stiffness that limit activities of daily living and independence.^17^ Many patients with thumb basal joint arthritis are amenable to nonsurgical management techniques aimed at relieving pain, improving strength and mobility, and restoring function. However, a certain subset of patients will require surgical intervention to address their symptoms. The literature approximates that 15% of patients who underwent conservative measures with hand therapy and orthotics required surgical treatment within just over 2 years.^18^ Importantly, a recent study highlights the impact of psychological factors on symptoms in patients with thumb CMC arthritis. Hoogendam et al^19^ reported that in patients with basilar joint arthritis, psychological factors explained nearly half of the variance in pretreatment pain, whereas radiographic severity and patient characteristics accounted for only 6%.

Although MDD has been shown to negatively affect outcomes of various orthopedic surgical procedures, its impact on patients undergoing thumb CMC arthroplasty remains understudied. Understanding the effect of depression and antidepressant use on patient-reported outcomes in patients undergoing trapeziectomy and ligament reconstruction tendon interposition (LRTI) surgery for thumb CMC arthritis may help guide both patient and provider expectations regarding outcomes after surgery. It may also elucidate whether there are any psychological or pharmacological protective effects for patients during the recovery period. We hypothesize that patients with a diagnosis of MDD or active antidepressant treatment before surgery will have poorer upper-extremity outcomes at 1 year compared with patients without MDD or antidepressant treatment.

## Materials and Methods

### Study design

This study was carried out at a single academic medical center following approval from the institutional review board. We reviewed patients who underwent thumb carpometacarpal (CMC) arthroplasty for basilar thumb arthritis between January 2005 and December 2023. All procedures involved a trapeziectomy with flexor carpi radialis ligament reconstruction and tendon interposition, performed by 2 fellowship-trained hand surgeons. Inclusion criteria were patients aged 18 years or older who underwent an isolated CMC arthroplasty for primary osteoarthritis with a minimum of 1 year follow-up. Patients with other chronic upper-extremity conditions (such as inflammatory arthritis, cervical radiculopathy, or prior trauma to the operative limb) or those who underwent concurrent procedures at the time of CMC arthroplasty were excluded. Patients with psychiatric diagnoses other than MDD, including generalized anxiety disorder, bipolar disorder, obsessive-compulsive disorder, posttraumatic stress disorder, and psychotic disorders, were also excluded.

Eligible patients were identified using Current Procedural Terminology codes 25210 and 25447 for carpectomy and interposition arthroplasty of the CMC joint, respectively. Only patients treated with trapeziectomy and LRTI using the flexor carpi radialis were included.

To assess the impact of depression, patients were identified based on a documented diagnosis of MDD or active treatment with antidepressant medications at the time of surgery (we will refer to this cohort as “MDD”). Antidepressant medications include selective serotonin reuptake inhibitors, serotonin-norepinephrine reuptake inhibitors, tricyclic antidepressants, bupropion, and monoamine oxidase inhibitors. Medications such as anxiolytics or antipsychotics, often used for off-label indications, were not classified as antidepressants for this study. If a patient was prescribed antidepressants but did not have a clearly documented diagnosis of MDD, their records underwent an additional manual review, including pharmacy claim verification, and they were classified in the depression group (despite the lack of a formal diagnosis in their medical record). The remaining patients without MDD or antidepressant use formed the comparison group for this study (we will refer to this cohort as “CONT”).

### Data collection

Collected variables included demographic characteristics (age, sex, gender, race, ethnicity), insurance type (private, Medicare, Medicaid, Workers’ Compensation), and clinical information such as indication for surgery, body mass index (BMI) at the time of surgery, comorbidities, hand dominance, and smoking status. We also recorded the Eaton classification as documented by the attending surgeon based on their independent review of preoperative radiographs on initial consultation. Operative details, postoperative course, and complications (from the time of surgery to present), including reoperations, subsidence, and infections, were recorded.

Patient-reported outcomes were evaluated using the Quick Disabilities of the Arm, Shoulder, and Hand (*Quick*DASH) survey. The *Quick*DASH survey, an 11-item questionnaire, is used to measure disability/symptom severity for patients with upper-extremity disorders, with scores ranging from 0 (no disability) to 100 (most severe disability). This was administered at baseline (before surgery) and during follow-up visits at 3, 6, and 12 months after surgery.

### Statistical analysis

Power analysis demonstrated that with complete data from 152 patients, the study would have 80% power at 2-sided alpha of 0.05 to detect a difference as low as 10 points in the unadjusted *Quick*DASH score change from baseline to 12 months between the MDD and CONT groups. This assumes a ratio of 1:3 between the MDD and CONT groups and a correlation between the baseline and 12-month *Quick*DASH score of 0.5. Given that the *Quick*DASH has a minimal clinically important difference of 14 points,^20^ the study would have enough power to detect important differences in *Quick*DASH change between the 2 groups. Accounting for a loss to follow-up of 50%, the study would need to analyze data from at least 304 patients.

We generated descriptive statistics (mean and standard deviation for continuous variables and absolute and relative frequencies for categorical variables) to characterize our sample. *Quick*DASH scores measured over time (before surgery, 3, 6, and 12 months after surgery) were summarized by calculating the average and standard deviation for the entire sample and by group (MDD, no MDD). We created graphical representations for the trajectory of *Quick*DASH scores from baseline to 12 months after surgery using R software.^21^

We used the paired samples *t* test for the improvement in *Quick*DASH scores from baseline to 12 months in each group and the independent samples *t* test to test for differences in *Quick*DASH score change (baseline to 12 months) by the presence of MDD. Further, we implemented a linear mixed effects model to estimate the differences in *Quick*DASH scores by the presence of MDD while adjusting for the time from surgery, sex, race, BMI at the time of surgery, and the interaction between MDD diagnosis and time from surgery. We also explored whether the BMI at the time of surgery, age, gender, or race affected the recovery, as measured by the *Quick*DASH scores over time. To test this, we included an interaction term with time for each of these variables and retained in the model depending on the significance. In addition to running the analysis based on the linear mixed effects model, we verified the model assumptions. To address the missing data issue, we implemented multiple imputation, which is a statistical technique that, based on the assumed missing data mechanism, creates multiple complete data sets.^22^ We implemented this technique using PROC MI of the SAS software, version 9.4, with fully conditional specification by predictive mean matching, which resulted in 30 complete data sets.^23^ After creating the imputed data sets, we implemented the mixed models to estimate the differences in *Quick*DASH. Further on, we used PROC MIANALYZE to apply Rubin’s rules to pool the results obtained from the mixed models across the 30 imputed data sets and calculate the coefficient estimates and the corresponding standard errors.^22^

## Results

A total of 328 patients met the inclusion criteria. Among them, 22.3% had a confirmed diagnosis of MDD or were taking antidepressants (MDD group). The mean age of the cohort was 63.8 years (SD ± 8.5), and most patients were women (69.3%) and White (88.3%) (Table 1). Of all the patients, 6.2% identified as current smokers, 34.9% as former smokers, and 59.0% reported never having smoked. Insurance coverage included 56.1% patients with commercial insurance, 43.6% with Medicare, 15.8% with Medicaid, and 3.4% with Workers’ Compensation.

**Table 1:** Patient Demographics (N = 328)

Characteristic	Frequency (%)
Age (y)	63.8 ± 8.5
Sex	
M	100 (30.7)
F	226 (69.3)
*Missing*	*2*
Race	
White	287 (88.3)
Black	19 (5.8)
Asian	4 (1.2)
Hispanic	4 (1.2)
Refused to answer	11 (3.4)
*Missing*	*3*
Ethnicity	
Hispanic	16 (5.1)
Non-Hispanic	296 (94.9)
*Missing*	*16*
Smoking status	
Current	20 (6.2)
Former	113 (34.9)
Never	191 (59.0)
*Missing*	*4*
Health insurance	
Medicare	143 (43.6)
Medicaid	19 (5.8)
Commercial	184 (56.1)
Workers	11 (3.4)
Others	5 (1.5)
*Missing*	*12*
Diagnosed with MDD prior to surgery	
Yes	73 (22.3)
No	255 (77.7)

Average preoperative *Quick*DASH scores were 41.1 for the MDD group and 40.3 for the CONT group. When examining the trends over the postoperative time course, *Quick*DASH scores improved at each time point in both groups, achieving their lowest numbers at 1 year after surgery. At 3 months, *Quick*DASH scores were 34.7 for the MDD group and 35.4 for the CONT group. At 6 months, *Quick*DASH scores were 36.6 for the MDD group and 32.0 for the CONT group. At 12 months after surgery, *Quick*DASH had decreased to 28.8 and 24.0 for the MDD and CONT groups, respectively (Fig.), with the paired sample *t* test also showing that the decrease in *Quick*DASH scores from baseline to 12 months after surgery was statistically significant (*P* < .001) for both groups. However, there was no statistically significant difference in *Quick*DASH change from 0 to 12 months (*P* = .52). The number of missing data points was 72 at 3 months, 188 at 6 months, and 181 at 1 year.

**Figure:**
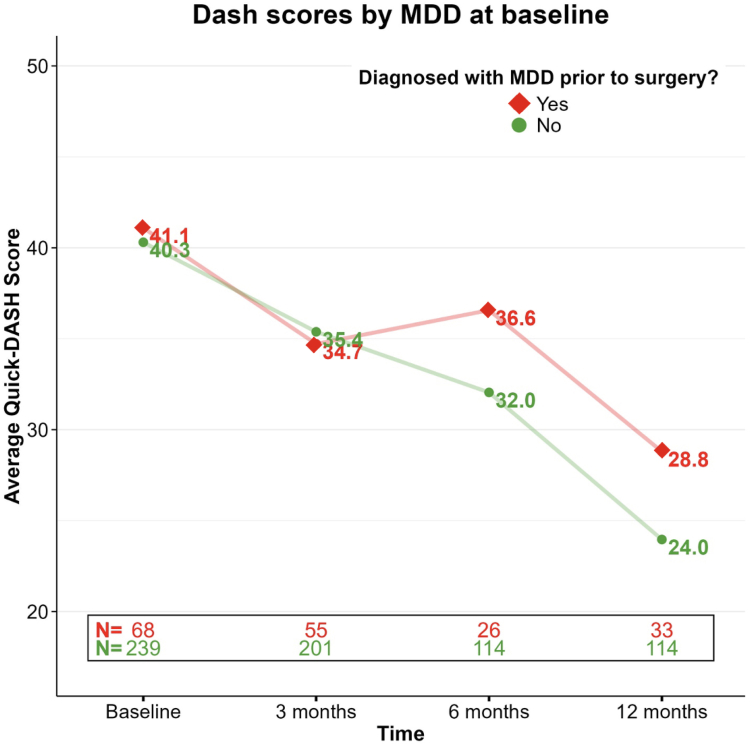
Comparison of *Quick*DASH scores from baseline to 1 year after surgery following CMC arthroplasty between patients diagnosed with MDD and those without an MDD diagnosis.

We then used the linear mixed model to assess the relationship between the presence of MDD at baseline and *Quick*DASH scores, adjusting for clinically and statistically important covariates. The model showed that for each month following the surgery, *Quick*DASH scores decreased by an average of 1.23 points (*P* < .001), indicating progressive improvement in patient-reported outcomes across the entire cohort (Fig.).

Patient race was found to be a significant predictor of patient-reported outcomes. White patients had *Quick*DASH scores that were, on average, 8.67 points lower than non-White patients (*P* < .01) at baseline and after surgery (Table 2). When considering sex differences, there was no significant difference in *Quick*DASH scores between men and women (*P* = .095). Age and BMI at the time of surgery did not demonstrate a statistically significant association with *Quick*DASH scores at any time point (*P* = .80 and *P* = 0.11, respectively).

**Table 2:** Patient Factors (MDD Diagnosis, Age, Sex, Race, BMI, and Time From Surgery) Associated With Overall Postoperative *Quick*DASH Scores: a Linear Mixed Model Analysis (N = 328)

Patient factor	B (SE)	*P* value
Diagnosed with MDD prior to surgery∗		
Yes	−0.10 (2.45)	.968
Age	0.04 (0.11)	.742
Sex†		
M	−3.67 (1.96)	.062
Race‡		
White	−9.47 (3.09)	.003
BMI at the time of surgery	0.25 (0.18)	.166
Time from surgery in months	−1.24 (0.13)	<.001
Interaction		
MDD diagnosis × time from surgery	0.25 (0.34)	.467

β coefficient represents the effect on *Quick*DASH scores (ie, increased or decreased number of points) associated with each corresponding predictor variable.

B (SE), β coefficient (standard error).

∗ Reference category is “No.”

† Reference category is “female.”

‡ Reference category is “non-White.”

Radiographic severity of thumb CMC arthritis was assessed using the Eaton classification system. Across the total cohort, most patients presented with Eaton grade 3 arthritis (73.5%), followed by grade 4 (18.0%) and grade 2 (6.1%). When stratified by depression status, there were no significant differences (*P* = .14 from Fisher’s exact test) in Eaton grade distributions between the 2 groups. Among CONT patients with MDD, 76.5% had grade 3 arthritis, compared with 71.0% of those with MDD. Grade 4 disease was observed in 18.7% of CONT patients and 17.4% of MDD patients. Grade 2 disease was observed in 4.8% of CONT patients and 11.6% of the MDD patients (*P* = .10). Mean Eaton grade was similar between groups (3.1 ± 0.5 for CONT vs 3.0 ± 0.5 for MDD), with a median of 3.0 in both groups.

Eleven patients (3.4%) had 1 relevant postoperative complication. Complications included superficial skin infection (N = 3), local skin reaction to suture/bandage (N = 4), wound dehiscence (N = 1), deep infection requiring surgical irrigation and debridement (N = 1), and extensor tendon subluxation (N = 2). There was no statistically significant difference (*P* = .72) in the rate of postoperative complications between the patients diagnosed with MDD and CONT (n = 3.9 and n = 3.2%, respectively).

## Discussion

In this study, we evaluated the impact of MDD and antidepressant use on patient-reported outcomes following trapeziectomy LRTI for thumb CMC arthritis. Despite the known burden of depression on recovery in other orthopedic populations,^6^^,^^7^^,^^24^, ^25^, ^26^ we did not find a statistically significant difference in *Quick*DASH scores at any postoperative time point between patients with and without MDD or active antidepressant use. Our findings suggest that while depression is a serious comorbidity, its influence on patient-reported disability after CMC arthroplasty may be less pronounced than anticipated or potentially mitigated by appropriate mental health treatment.

Considering the effects of having a known MDD diagnosis and current treatment regimen is imperative to analyzing the outcomes of our study. All patients included in the MDD group were actively receiving treatment or diagnosed with MDD before surgery, representing a subgroup of individuals whose depression was presumably under clinical management. A history of appropriate clinical care for MDD (ie, therapy, medications, or both) may explain why, despite having an MDD diagnosis, there was no significant difference in the trajectory of improvement in *Quick*DASH scores and outcomes. This overall trajectory of improvement was consistent with prior work evaluating patient-reported outcomes following CMC arthroplasty.^27^ Cochrane et al^27^ evaluated the utility of screening for depressive symptoms using the PROMIS depression score in Hand surgery patients. They found that although patients with diagnosed MDD continued to report more symptoms than unaffected individuals, those with MDD receiving antidepressants had a 4-point improvement in their PROMIS score compared with untreated MDD patients, which was their threshold for clinical relevance. This supports the notion that treatment may mitigate the negative impact of MDD on surgical recovery. Notably, in the same study, 7% of patients without a diagnosis screened positive for heightened depressive symptoms.^27^ The under-recognized burden of depression suggests that undiagnosed or untreated cases in the non-MDD group may weaken the differences between groups and contribute to the lack of statistical significance in reported outcomes.

The relationship between MDD and poorer upper-extremity outcomes has often been reported in the posttraumatic setting,^13^^,^^15^^,^^24^ where patients are simultaneously coping with pain, physical injury, loss of function, and psychosocial stressors. These patients are often dealing with a sudden interruption in employment, loss of income, or independence in activities of daily living. Conversely, thumb CMC arthroplasty is an elective intervention for a chronic degenerative condition, typically performed in patients who have had time to adapt to functional limitations, prepare for surgery, and engage in preoperative counseling with their surgeon. This anticipatory framework may ease the psychological stress of undergoing surgery and its associated recovery. The elective setting also allows patients with MDD to develop coping strategies and social support more effectively, as well as provides a more structured timeline for treatment of their mental health conditions (pharmacologic, psychosocial, or both). Prior findings by Bot et al^13^ have shown that such coping strategies, particularly higher self-efficacy, are strongly linked to less disability, pain, and stiffness after hand injuries. Thus, the absence of a traumatic episode and its associated acute stress reaction or sudden psychosocial disruption may partly account for the similarity in *Quick*DASH trends in the MDD and CONT cohorts, particularly when comparing our findings to the current literature.

When evaluating patient race, our results indicate that there is a difference in *Quick*DASH scores, with the *Quick*DASH score being on average 9.47 lower in White patients compared to non-White patients; however, the minimal clinically important difference for thumb CMC arthroplasty is 14.^20^ Although this difference may not represent a meaningful threshold change, it warrants further investigation. This may reflect differences in access to care, cultural attitudes toward surgery and pain, or unmeasured social determinants of health. It also emphasizes the importance of interpreting surgical care and postoperative recovery in the context of a patient’s broader sociodemographic environment.

Our study has several limitations. First, the retrospective design naturally restricts causal inference, and although we employed statistical methods to account for this limitation, residual confounding might still exist. Second, data on MDD diagnosis or antidepressant use were collected through chart reviews from our institution’s medical records, which may overestimate or underestimate the true prevalence of MDD because of incomplete records. Even though antidepressant prescriptions and use were reviewed, we could not verify adherence to treatment. Moreover, we did not include antipsychotics and other off-label medications; thus, we possibly failed to capture a subset of patients taking these medications for depression. Third, although the typical demographic of patients undergoing thumb CMC arthroplasty are White and women,^28^, ^29^, ^30^ which is consistent with the cohort in this study, the generalizability of our results to broader populations remains limited and warrants future research. Finally, the race difference noted when comparing baseline *Quick*DASH scores may be limited given the overall smaller sample size of non-White patients included in the study (12.1%).

Despite these limitations, our study contributes valuable insight into the relationship between MDD and surgical outcomes in a population for whom this association has been largely understudied. The absence of a strong adverse effect of depression on postoperative patient-reported outcomes after thumb CMC arthroplasty suggests that, with appropriate mental health support, patients with MDD can expect similar functional recovery to those without depression. These findings highlight the importance of a multidisciplinary approach that considers all aspects of patients’ health, not solely their orthopedic diagnosis.

## Conflicts of interest

Dr. Rozental is a consultant for Stryker and Teladoc Health, Inc. No benefits in any form have been received or will be received by the other authors related directly to this article.
